# Lectotypification of the name *Melastoma candidum* f. *albiflorum* and its taxonomic status

**DOI:** 10.3897/phytokeys.146.49929

**Published:** 2020-05-08

**Authors:** Xinjian Zhang, Jin-Hong Dai, Xiaozhou Liu, Zihua Li, Shiou Yih Lee, Renchao Zhou, Guangwen Tan

**Affiliations:** 1 Pubang Landscape Architecture Co., Ltd, Guangzhou 510600, China Pubang Landscape Architecture Co., Ltd Canton China; 2 State Key Laboratory of Biocontrol and Guangdong Key Laboratory of Plant Resources, School of Life Sciences, Sun Yat-sen University, Guangzhou 510275, China Sun Yat-sen University Guangzhou China

**Keywords:** flora of China, *
Melastoma
*, synonym, taxonomy

## Abstract

A nomenclatural and taxonomic treatment of the name Melastoma candidum f. albiflorum (Melastomataceae) is presented. A lectotype is designated for this name, with an updated morphological description based on fresh material. The name Melastoma candidum f. albiflorum is proposed as a heterotypic synonym of *Melastoma candidum*.

## Introduction

*Melastoma* L. comprises species mainly distributed in the Southeast Asia and extends to India, South China, Japan, northern Australia, and Oceania (Meyer 2001). *Melastoma* is taxonomically difficult, and the recognized number of species in this genus remains controversial due to rapid adaptive radiation and extensive natural hybridization (Renner and Meyer 2001; Liu et al. 2014; Zhou et al. 2017). It was claimed that this genus comprises about 100 species (Chen 1984), but only 22 species were recognized in the recent taxonomic revision (Meyer 2001; Chen and Renner 2007). However, it was estimated that this genus might include 80–90 species based on a scientific investigation of the island of Borneo (Wong 2016).

In China, nine *Melastoma* species were recorded in the south of the Yangtze River by Chen (1984), yet only five of them were recognized in more recent publications (Meyer 2001; Chen and Renner 2007). This revised treatment has incorporated *M. affine*, *M. normale* and *M. candidum* into *M. malabathricum*, but this has not been accepted by several plant taxonomists (Huang et al. 2018).

In this study, we are in agreement with the recognition of *M. candidum* as a distinct species (Liu et al. 2014; Ng et al. 2017). *Melastoma candidum* is a relatively common species with purple flowers that occurs in southern China and northern Vietnam (Chen 1984). Melastoma candidum f. albiflorum J. C. Ou, a form of *M. candidum*, was first described from Taiwan Island (Ou 1976). However, this name was not validly published under Art. 40 of ICN (McNeill 2014; Turland et al. 2018) because Ou (1976) cited two gatherings but failed to designate a type, and no Latin description or diagnosis was provided. This taxon can be easily distinguished from M. candidum f. candidum by its white flowers.

During a recent field survey, we collected an unidentified specimen of *Melastoma* in Fujian province, China, which we believed has not been recorded in mainland China. It closely resembles *M. candidum* in morphology, but has white flowers. After a comprehensive morphological comparison, we propose that this specimen be conspecific with M. candidum f. albiflorum. Here we designate a lectotype for this taxon name and discuss its taxonomic status.

## Materials and methods

Morphological data for identification and description of this taxon were based on observations of specimens in the field (ten individuals) and the herbarium. The voucher specimens are deposited in the Herbarium of Sun Yat-sen University (SYS), Guangzhou, China. Lectotypification and taxonomic treatment of this taxon is presented according to the "International Code of Botanical Nomenclature" (McNeill 2014; Turland et al. 2018), and a full description is provided.

## Results

**Figure 1.**
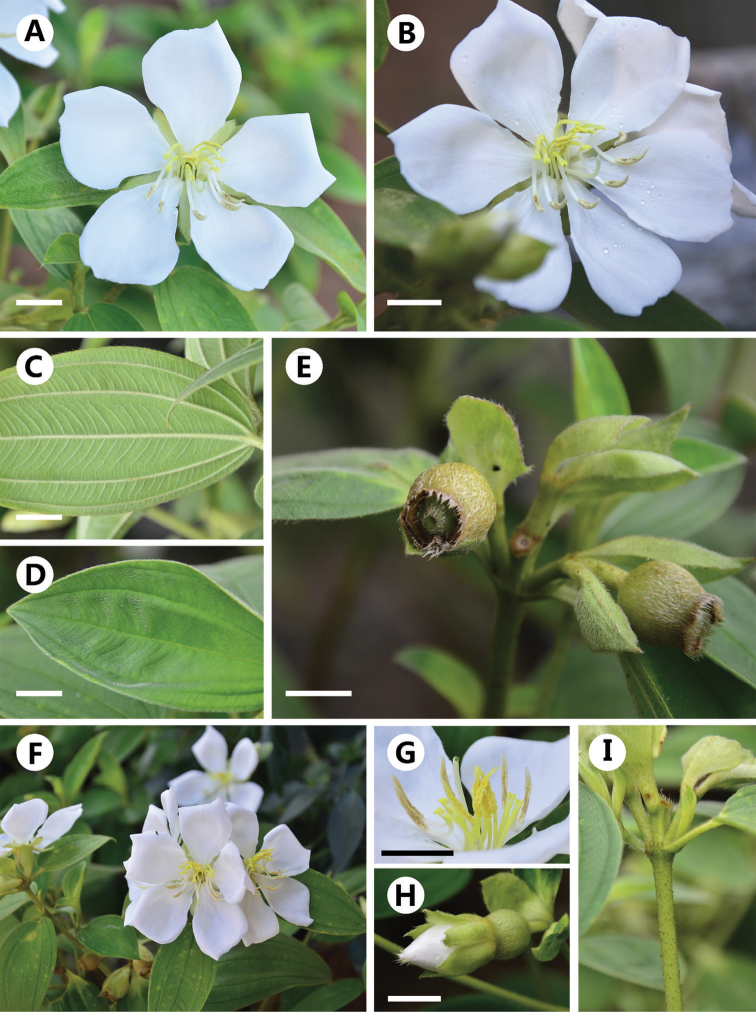
The white form of *Melastoma candidum*. **A** flower 5-merous **B** flower 6-merous **C** abaxial surface of leaf blade **D** adaxial surface of leaf blade **E** capsule **F** inflorescences **G** apex of flower showing heteranthery **H** close-up of bud showing margin ciliate of sepals and petals **I** twig of plant with pedicels Scale bars: 1 cm (**A–E, G, H**).

Detailed morphological examination revealed that morphological characters of this unidentified taxon of *Melastoma*, such as erect habit, ovate leaves, twigs with compressed scales, hypanthia with compressed strigose scales, and fruits with densely appressed strigose scales (Fig. 1), are most similar to *M. candidum*. The two taxa differ only in petal color, with white petals in this taxon and purple petals in *M. candidum*. Considering that this taxon is sympatric with *M. candidum* in Fujian and Taiwan, we propose that M. candidum f. albiflorum is conspecific with and should be synonymized under *M. candidum*.

### Taxonomic treatment

#### Melastoma candidum

Taxon classificationPlantaeMyrtalesMelastomataceae

D. Don

9C364FE8-C9E6-5101-8F6F-216089BDB8D6

##### Synonym.

Melastoma candidum D. Don f. albiflorum J. C. Ou.

##### Lectotype

(designated here): – China. Taiwan. Ilan, Yuensanhsiang, leg. *Jun-Chih Ou 64*, July 6, 1976, Herbarium of National Research Institute of Chinese Medicine (HNRICM!).

##### Description.

Perennial shrubs, 0.3–1.5 m tall. Twigs nearly 4-angled to subterete in the younger parts and terete in the old parts, densely covered with appressed to suberect strigose with scales. Leaf blades ovate to elliptic, papery, 3.3–4.8×6–9.5 cm, base broadly cuneate to rounded or subcordate, apex acuminate, margin entire, palmately 7-nerved (the marginal nerves often inconspicuous), adaxially densely strigose, abaxially densely puberulous, strigose along veins; petioles 1.4–2.0 cm, densely strigose with scales. Inflorescences subcapitate corymbose, terminal, 3–5-flowered, with 2 leaf-like bracts at base. Pedicels 8–12 mm, densely strigose with scales; bracteoles 2, opposite, elliptic-lanceolate to elliptic, 6–13 mm, abaxially densely strigose, margin ciliate. Hypanthia 7–12 mm, densely appressed-strigose with scales, margin fimbriate. Sepals lanceolate to ovate-lanceolate, apex acuminate, densely strigose and pubescent on both sides and along the margin. Petals 5, occasionally 6, white, obovate, ca. 27×18 mm, apex rounded. Stamens 10, dimorphic, longer stamens with anthers linear, curved, ca. 9 mm, filaments ca. 10 mm, joined by a connective ca. 9 mm, curved, spur bifid ca. 2 mm, shorter stamens with anthers ca. 8 mm, 2-tuberculate at base, filaments ca. 7 mm, without prolonged connective. Ovary half-inferior, campanulate, with a ring of bristles at apex. Capsule dry, urceolate, apically dehiscent, 9–16×7–10 mm, densely squamose strigose. Seeds numerous, minute, cochleate. Fl. May-Aug, fr. Aug-Oct.

##### Distribution and habitat.

**Figure 2.**
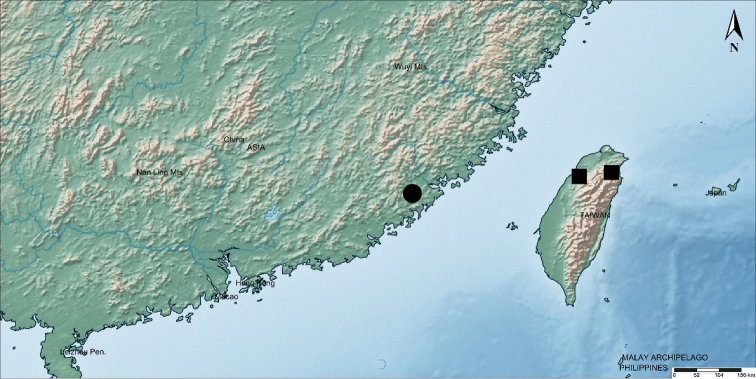
Distribution map of the white form of *Melastoma candidum*. Square (■) represents previously reported localities, solid circle (●) represents newly recorded locality. Map was created using SimpleMappr, http://www.simplemappr.net (Shorthouse 2010).

The white-flowered form of *M. candidum* was first reported only from Hsinchu Hsien and Ilan Hsien (Taiwan). This form has also been reported to occur in the Ryukyu Island (Hatusima and Amano 1994), but without exact specimen information. The individuals in Fujian, China represent the first known occurrence of this form outside of Taiwan Island (Fig. 2). It occurs only in lowland evergreen forest margins at an elevation of approximately 150–300 in Pinghe County, Fujian. They occur in evergreen forests dominated by *Blechnum orientale*, *Dicranopteris pedata*, *Miscanthus floridulus* and *Rhodomyrtus tomentosa*.

##### Notes.

The key character of the white form of *M. candidum* is its white-colored flowers, which can be easily distinguished from the purple form of *M. candidum*. Whereas the purple form has a relatively wide range of distribution in northern Vietnam and southern China (Liu et al. 2014), the white-flowered individuals have been found across a narrow region. Individuals in Taiwan and mainland China may represent independent origins of white petals from local populations of *M. candidum*, since breakdown of the anthocyanin synthesis pathway in plants is relatively common (Smith et al. 2012; Zheng et al. 2019).

The flowers of the white form of *M. candidum* have been used in folk-medicine for the treatment of hypertension, dysentery, diarrhea and antibacterial (Chou and Liao 1982). During our survey, we also learned that the white form has been cultivated as a medicinal herb by the local people in Pinghe County, Fujian. They believe that it is highly effective for the treatment for nephritis, and has led to the exploitation of natural populations, threatening its survival in the wild. Due to its narrow geographical range and small population size, effective conservation effort is required.

##### Paratype.

– China. Fujian Province, Pinghe County, in lowland evergreen forest margins, 24°02.66'N, 117°04.75'E, Elev. 276 m. 28 July 2019, X. J. Zhang, *ZXJ-1901* (SYS)

## Supplementary Material

XML Treatment for Melastoma candidum
